# Genomic dissection of the 1994 *Cronobacter sakazakii* outbreak in a French neonatal intensive care unit

**DOI:** 10.1186/s12864-015-1961-y

**Published:** 2015-10-05

**Authors:** Naqash Masood, Karen Moore, Audrey Farbos, Konrad Paszkiewicz, Ben Dickins, Alan McNally, Stephen Forsythe

**Affiliations:** Pathogen Research Group, School of Science and Technology, Nottingham Trent University, Clifton Lane, Nottingham, NG11 8NS UK; Wellcome Trust Biomedical Informatics Hub, Biosciences, Stocker Road, University of Exeter, EX4 4QD Exeter, UK

**Keywords:** *Cronobacter sakazakii*, Clonal complex, MLST, Neonatal meningitis, SNP analysis

## Abstract

**Background:**

*Cronobacter sakazakii* is a member of the genus *Cronobacter* that has frequently been isolated from powdered infant formula (PIF) and linked with rare but fatal neonatal infections such as meningitis and necrotising enterocolitis. The *Cronobacter* MLST scheme has reported over 400 sequence types and 42 clonal complexes; however *C. sakazakii* clonal complex 4 (CC4) has been linked strongly with neonatal infections, especially meningitis.

There have been a number of reported *Cronobacter* outbreaks over the last three decades. The largest outbreak of *C. sakazakii* was in a neonatal intensive care unit (NICU) in France (1994) that lasted over 3 months and claimed the lives of three neonates. The present study used whole genome sequencing data of 26 isolates obtained from this outbreak to reveal their relatedness. This study is first of its kind to use whole genome sequencing data to analyse a *Cronobacter* outbreak.

**Methods:**

Whole genome sequencing data was generated for 26 *C. sakazakii* isolates on the Illumina MiSeq platform. The whole genome phylogeny was determined using Mugsy and RaxML. SNP calls were determined using SMALT and SAMtools, and filtered using VCFtools.

**Results:**

The whole genome phylogeny suggested 3 distant clusters of *C. sakazakii* isolates were associated with the outbreak. SNP typing and phylogeny indicate the source of the *C. sakazakii* could have been from extrinsic contamination of reconstituted infant formula from the NICU environment and personnel. This pool of strains would have contributed to the prolonged duration of the outbreak, which was up to 3 months. Furthermore 3 neonates were co-infected with *C. sakazakii* from two different genotype clusters.

**Conclusion:**

The genomic investigation revealed the outbreak consisted of an heterogeneous population of *C. sakazakii* isolates. The source of the outbreak was not identified, but probably was due to environmental and personnel reservoirs resulting in extrinsic contamination of the neonatal feeds. It also indicated that *C. sakazakii* isolates from different genotype clusters have the ability to co-infect neonates.

**Electronic supplementary material:**

The online version of this article (doi:10.1186/s12864-015-1961-y) contains supplementary material, which is available to authorized users.

## Background

*Cronobacter sakazakii,* a member of the genus *Cronobacter* is linked with serious infections such as meningitis, septicaemia and necrotizing enterocolitis (NEC) in neonates and immunocompromised adults [1–6]. The organism has been isolated from powdered infant formula (PIF), and various environmental sources including hospital air, dust, formula preparation areas and equipment [7–9]. Human carriage has been reported in saliva, teeth, faeces, breast milk, and skin [10, 11]. The first genome sequenced strain of *C. sakazakii* was associated with a NICU outbreak at the University of Tennessee Hospital [2, 12]. According to pulsed-field gel electrophoresis analysis, this strain was recovered from an infant who had died from meningitis, two further suspected infections (tracheal aspirate positive samples), and seven neonates who were asymptomatically colonised (six faecal positive and one urine positive isolations), therefore reflecting the dispersal of the strain within the NICU.

There have been other reported outbreaks of *C. sakazakii* [5], and one of the largest was in a NICU in France claiming the lives of at least 3 neonates [1]. The outbreak lasted for approximately 3 months (111 days; 5 May 1994 to 11 July 1994). A total of 18 neonates were infected or colonized with *C. sakazakii*, and 3 died. Neonate H died of meningitis while neonates J and F died of NEC. Most of the neonates were under-weight with an average weight of 1461 g. With the exception of neonate D, all of the neonates were delivered pre-term. An autopsy of neonate H who died of meningitis revealed cerebral lesions. All of the infected neonates developed clinical symptoms within 28 days of birth, with the exception of neonate K who developed symptoms of NEC 78 days after birth. All neonates (F, H, J) who died were low-weight with weights of 1000, 1500 and 1560 g, respectively. Four of the neonates (C, E, O, Q) were asymptomatically colonized while only 2 of the neonates (N and P) developed digestive problems of moderate nature. Genotypic and phenotypic analysis of the *C. sakazakii* strains isolated from this outbreak has previously been undertaken by Caubilla-Barron [1], however their study was focused on the diversity of the isolated strains and was not primarily an epidemiological investigation. Their pulsed-field gel electrophoresis (PFGE) analysis divided all strains into four distant pulsotypes (PFGE 1–4) [1]. More than one *C. sakazakii* pulsotype was recovered from three neonates (B,C,D).

It is notable that all three fatalities during this outbreak were attributed to the pulsotype two cluster and belong to clonal complex 4 (CC4) of the *Cronobacter* MLST scheme (http://pubmlst.org/cronobacter/) [2, 13]. Previous studies by our group have indicated a strong association of *C. sakazakii* CC4 with neonatal meningitis [3, 4] as well as being up to 25 % of strains isolated from milk powder and infant formula manufacturing plants [14].

Although PFGE is still widely used in outbreak analysis, it has several limitations, for instance co-migration of the similar sized bands can obscure their discrimination and some strains do not give banding patterns [15]. Single Nucleotide Polymorphism (SNPs) can cause significant changes in phylogenetic distances but they may not change the PFGE pattern. Therefore isolates may appear identical on PFGE when they are not, and hence misinform an epidemiological data investigation [15]. SNP analysis is being used to differentiate outbreak isolates which often show very low sequence diversity, such as the outbreak of *E. coli* O157:H7 attributed to salad bar and romaine lettuce contamination [15, 16].

The present study focused on a detailed genomic analysis of the *C. sakazakii* strains using SNP analysis to reveal the relatedness of the isolates, which may guide improved epidemiological studies in the future. *C. sakazakii* strains previously analysed using PFGE by Caubilla-Barron et al. [1] were sequenced using the Illumina MiSeq platform.

## Results and discussion

### Core genome phylogeny

A maximum likelihood (ML) phylogeny of the 26 *C. sakazakii* strains was constructed from a core genome alignment using Mugsy [17]. The topology of the phylogeny was in agreement with the earlier PFGE profiles [1]. Unfortunately, due to the long gap between the outbreak and present study, two of the isolates (704 of PFGE 1 and 697 of PFGE 2) have been lost from the culture collection, however the absence of these strains did not significantly affect the in-depth analysis of this outbreak at the genomic level presented in this study.

**Fig. 1:**
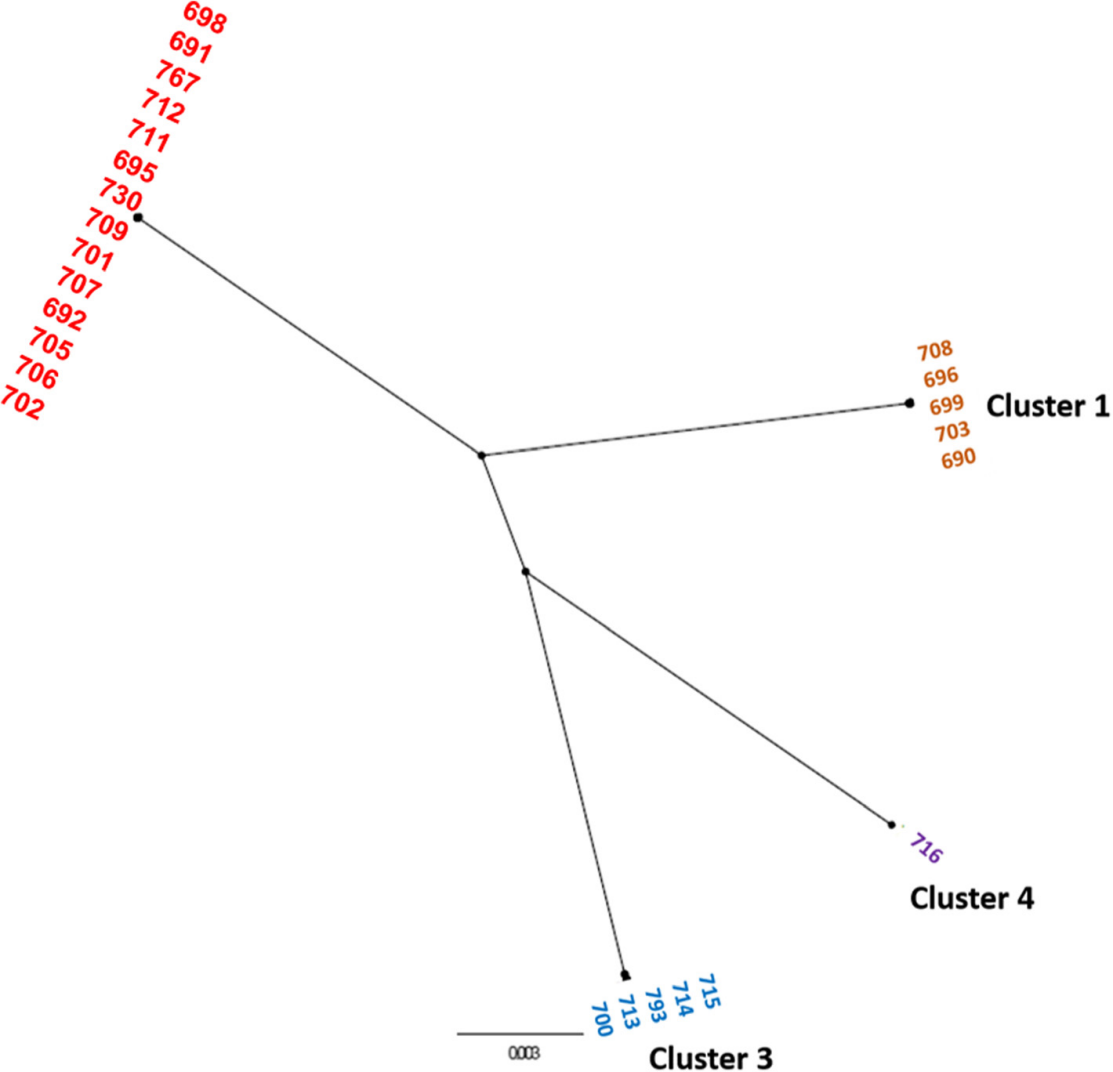
Un-rooted radial whole genome maximum likelihood tree of 26 *C. sakazakii* strains isolated from French NICU outbreak, 1994. The genomes of 26 C. sakazakii strains approximately 4 million bp were aligned using Mugsy, core genome extracted and maximum likelihood phylogeny generated using RAxML using GTRGAMMA substitution model. The tree was viewed and annotated using FigTree and Microsoft PowerPoint (version 2013). The phylogeny indicated 4 distant clusters. Cluster 1: ST12 and PFGE1 (orange font colour), Cluster 2: CC4 and PFGE2 (red font colour), Cluster 3: CC13 and PFGE 3 (blue font colour), Cluster 4: CC1 and PFGE4 (purple font colour). The scale bar indicates the rate of nucleotide differences per sequence site

The ML phylogeny indicated four clusters within these strains, cluster 2 being the largest group (Fig. 1). Three of the clusters (1, 2, 3) were isolated from neonates while the fourth consisted of a lone strain isolated from an un-opened can of infant formula. The result of the whole genome phylogeny is in complete agreement with the original PFGE profiles [1]. Since the grouping of the strains was identical to the PFGE profile, for clarity the clusters were given the same numbers as their earlier pulsotypes. It was interesting to note that all the strains within cluster 2 (equivalent to pulsotype 2) belong to *Cronobacter* MLST clonal complex (CC) 4 which has been associated predominantly with neonatal meningitis [3, 4] and all three neonates who died were infected by the genotype cluster 2 *C. sakazakii* CC4 strains [1].

### Single nucleotide polymorphism analysis

The SNP analysis was performed only on strains in clusters 1 to 3 as the genome sequence of only a single strain from cluster 4 was available (Table 1). Using an un-related reference genome can significantly increase the number of SNPs which ultimately can affect the SNP phylogeny [15]. Therefore in the present study each cluster was analysed independently of the other clusters. The earliest isolate in each cluster was used as the index strain to identify SNPs in the subsequently isolated strains. To improve the quality of identified SNPs, the reference genome assembly was improved using PAGIT; for this purpose the finished genome of *C. sakazakii* SP291 (ST4) was used as the reference genome for ordering the contigs. The SNP calls were determined using SMALT and SAMtools as described previously. The SNP phylogenies were constructed using the alignment of concatenated SNP profile in each cluster, hence there were variable substitutions/nucleotide as each cluster represented a different sequence type/clonal complex.

**Table 1:** Sequenced isolates of *C. sakazakii* French NICU outbreak 1994 and SNP typing

*C. sakazakii* strain	Genbank accession	Pulsotype	Sequence type^a^	Neonate	Symptoms	Isolation date (1994)	Day	Isolation site	SNP differences from index strain
699	JOLK00000000	1	12	A	No details	23-Mar	1	Trachea	Index strain
703	JOLY00000000	1	12	B	NECII	25-Apr	34	Trachea	63
708	JOLZ00000000	1	12	C	No-symptoms	09-May	48	Trachea	52
696	JOLW00000000	1	12	D	NECII	08-Jun	78	Stools	14
690	JOLN00000000	1	12	E	No-symptoms	19-Jun	89	Stools	64
701	LCWW00000000	2	4	F	NEC III (died)	07-Apr	1	Peritoneal fluid	Index strain
691	JOLQ00000000	2	4	G	No details	19-Apr	12	Sputum	9
767	JNCX00000000	2	4	H	Meningitis (died)	11-May	34	Trachea	38
709	JOLJ00000000	2	4	C	Septicaemia	12-May	35	Trachea	13
705	JOLI00000000	2	4	B	NEC II	24-May	47	Trachea	339
695	JOLG00000000	2	4	J	NEC II (died)	07-Jun	61	Trachea	11
706	JOLH00000000	2	4	B	NEC II	09-Jun	63	Stools	304
692	JOLV00000000	2	4	L	NEC II	13-Jun	67	Stools	15
702	JOLF00000000	2	4	K	NEC I	13-Jun	67	Stools	10
694	JOLM00000000	2	4	M	NEC II	14-Jun	68	Conjunctivae	8
712	JOLE00000000	2	4			17-Jun	71	Prepared formula	6
707	JOLD00000000	2	4	B	NEC II	26-Jun	80	Skin	337
711	JOLC00000000	2	4	O	Asymptomatic	27-Jun	81	Stools	14
730	JOLT00000000	2	4	K	NEC I	27-Jun	81	Stools	10
698	JOLR00000000	2	4	D	NEC II	01-Jul	85	Stools	10
700	JOLL00000000	3	13	P	Digestive problems	15-Jun	1	Stools	Index strain
693	JOLO00000000	3	13	Q	Asymptomatic	18-Jun	3	Stools	4
713	JOLX00000000	3	13			20-Jun	5	End of bottle	4
714	JOLU00000000	3	13			27-Jun	12	End of bottle	3
715	JOLP00000000	3	13			27-Jun	12	Prepared formula	3
716	JOLS00000000	4	14			11-Jul		Infant formula	

^a^Forsythe et al. [2]

*NEC* necrotizing enterocolitis

### SNP typing of cluster 1

**Fig. 2:**
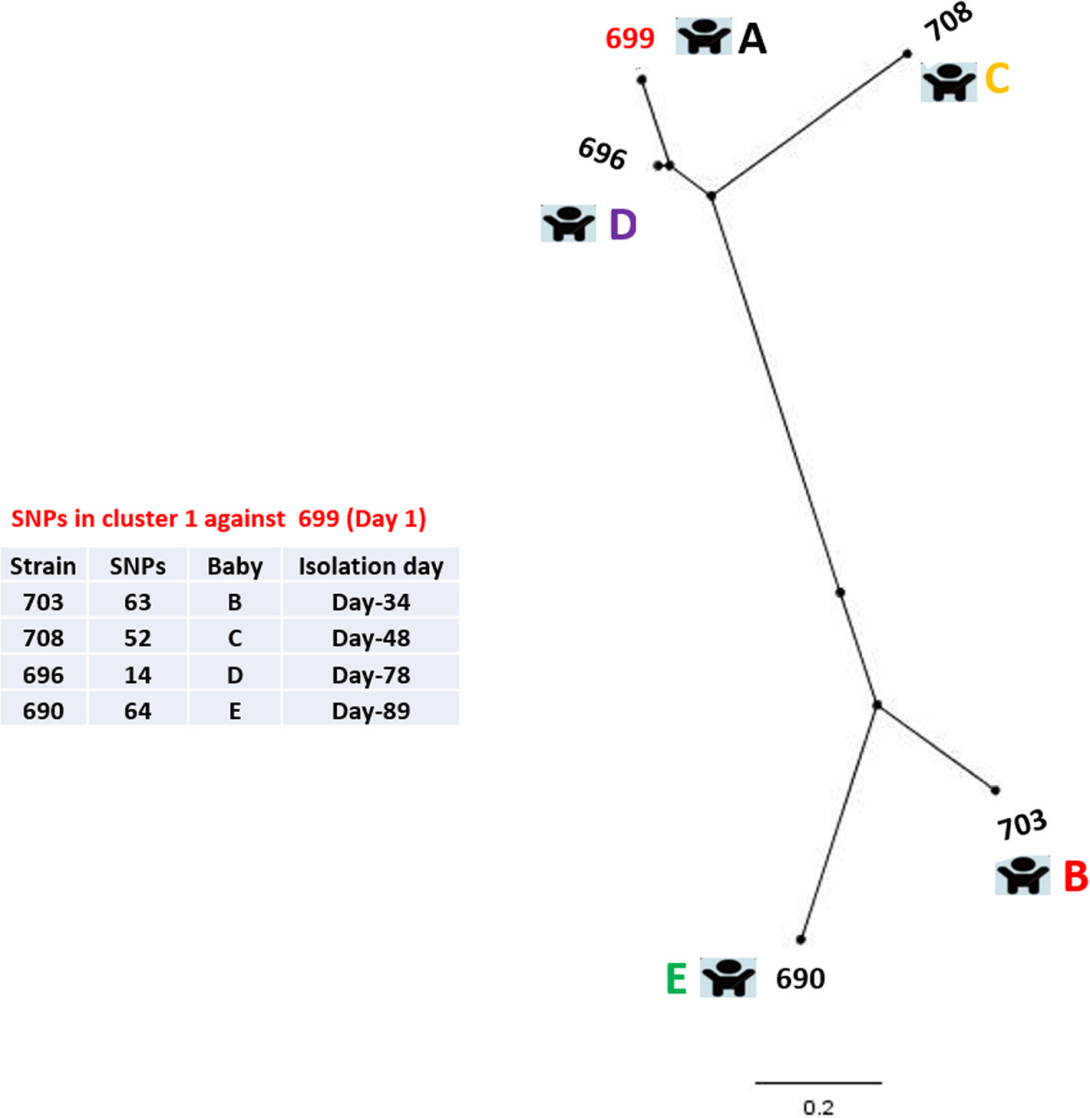
SNP phylogeny of the cluster 1 strains. The SNPs were called using SMALT and SAMtools to generate the VCF files which were filtered using VCFTools to include only SNPs with minimum quality score of 30, minimum depth of 8, and minimum allele frequency of 0.90. The SNPs in the cluster were concatenated and used to create a maximum likelihood phylogeny. The tree was rooted to midpoint. The red font colour indicates the reference strain. The scale bar indicates the rate of nucleotide differences per sequence site

Five of the isolates formed cluster 1 which were isolated between 23rd March and 19th June 1994 from neonates A, B, C, D and E (Table 1). All of the isolates in cluster 1 belong to *C. sakazakii* CC12 [2]. Two of the affected neonates (B and D) developed symptoms of NECII while 2 (C and E) remained asymptomatic, for the remaining neonate (A) no clinical details were available. *C. sakazakii* 699 was the earliest isolate of this cluster. It had been isolated from the trachea of neonate A on 23 March 1994 and was used as the index strain for SNP typing and SNP phylogeny determination (Fig. 2).

The phylogeny and the number of the SNPs indicated heterogeneity within cluster 1 isolates. The isolate 696 was more closely related to the index isolate 699 as they differ from each other only by 14 SNPs. The remaining three strains (703, 708 and 690) were relatively distant to the index strain by over 50 SNPs each. A likely scenario for the observed phylogenetic pattern is that all of the isolates obtained came from a common source where ongoing growth and evolution of *C. sakazakii* was occurring. These three strains could have been from earlier ingestion of contaminated reconstituted feed followed by colonisation and microevolution within the neonates before isolation. In this case, it is hard to predict the precise source of origin for the *C. sakazakii* isolates. *C. sakazakii* has the ability to survive diverse range of environments [7–11]. Therefore sources of contamination include the infant formula manufacturing plant or environment, the hospital environment, formula preparation area and equipment or hospital personnel.

The level of mutations observed of 14 SNPs in 78 days would be in accordance with postulated mutation rates occurring in hospital outbreaks or within patients [18]. The presence of an ongoing source would also presumably result in environmental contamination with a pool of bacterial diversity accumulating over time, which would also account for the diversity in the dozens of SNPs range seen here [19]. This is also supported by microbiome studies which have indicated the dispersion of bacteria in the NICU [20].

### SNP typing of cluster 2

**Fig. 3:**
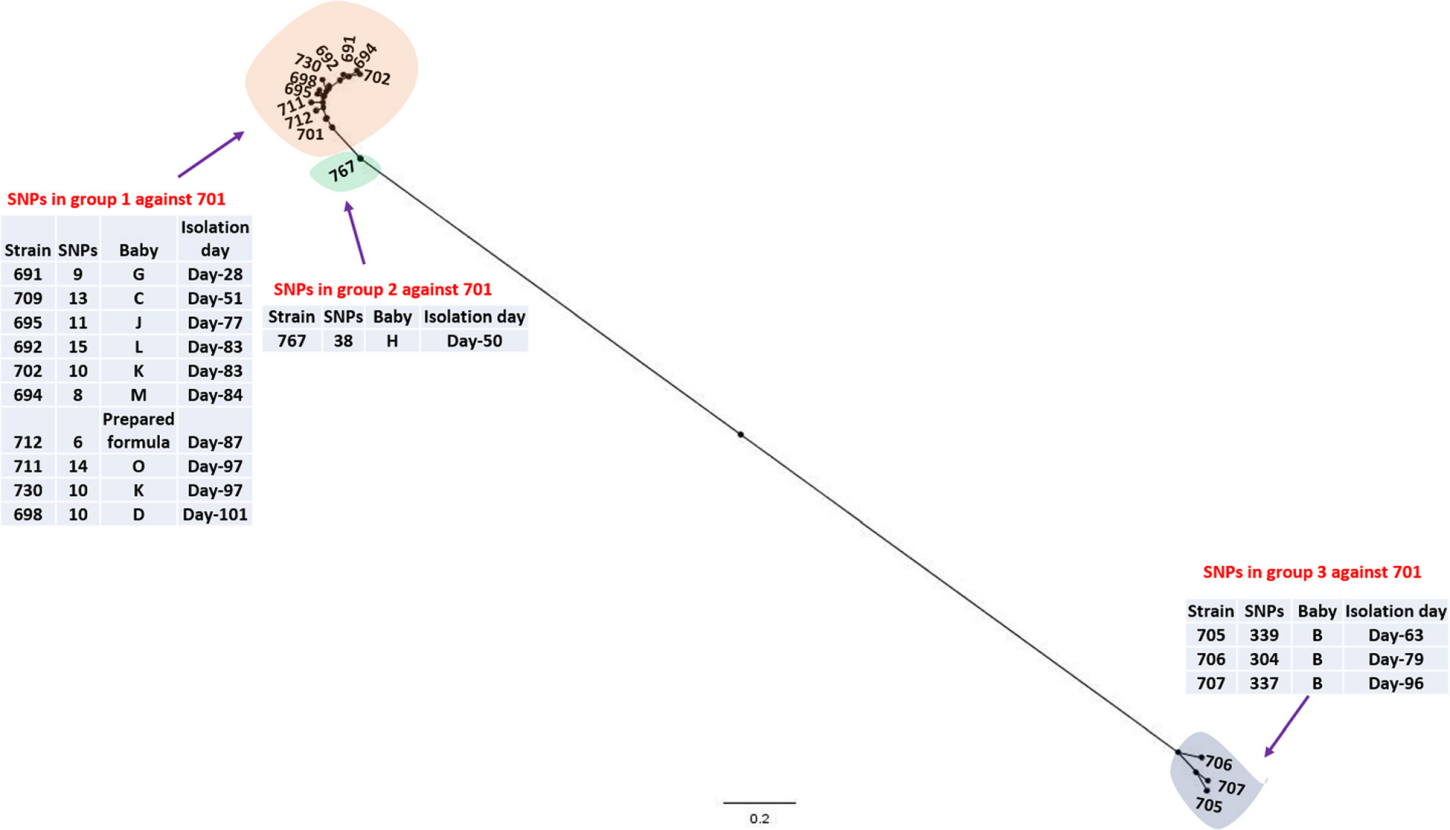
2SNP phylogeny of the cluster 2 strains. The SNPs calls were obtained using SMALT and SAMtools to generate the VCF files which were filtered using VCFTools to include only SNPs with minimum quality score of 30, minimum depth of 8, and minimum allele frequency of 0.90. The SNPs in the cluster were concatenated and used to create a maximum likelihood phylogeny. The tip labels shaded orange, green and yellow indicate groups 1, 2 and 3 respectively within cluster 2. The red tip label shows the reference strain. The tree was rooted to midpoint. The scale bar indicates the rate of nucleotide differences per sequence site

A total of 15 sequenced strains belonged to cluster 2 and had been isolated between 7th April and 1st July, 1994. All of the isolates in cluster 2 belong to *C. sakazakii* CC4 and were previously defined as pulsotype 2. These strains had been isolated from different neonatal sites including peritoneal fluid, sputum, trachea, stools, skin, and conjunctivae. The period of isolation of these strains overlapped with cluster 1 strains by 3 months. The 11 cluster 2 strains had been isolated from NEC cases at different stages, one from a septicaemia case and one from a meningitis case. The remaining two isolates were obtained from neonates O and G for whom no clinical details were provided. The earliest isolate of this cluster was *C. sakazakii* 701 isolated on 7th of April 1994. This strain was used as the index strain for SNP calls in order to observe the strain relatedness and possible route of transmission. The SNPs in each of the strains isolated according to the date order were determined using SAMTools (Table 1) and the SNP phylogeny (Fig. 3) was constructed as described earlier.

The tree topology indicated at least three subdivisions within the cluster 2 strains; a group of 11 isolates (group 1) joined by a short branch to isolate 767 (group 2) and through a longer branch with three isolates 705, 706 and 707 (group 3). All of the isolates in group 1 (Fig. 3, orange shaded) had a high degree of similarity with the index strain as shown by the small number of SNP differences; a maximum of 15 SNP differences to the index strain 701.

An interesting observation was the close relatedness of the index isolate 701 with the prepared formula isolate 712 which differed by only 6 SNPs. Although the index strain 701 was isolated at least 2 months earlier than the prepared formula isolate 712, the tree topology and small number of SNP differences suggest a possible common source of origin. The source could either be intrinsic contamination followed by growth in the neonate, or extrinsic sources such as the water used to prepare formula, colonization of feed preparation utensils or personnel. Two of the isolates (702 and 730) were obtained from the faeces of neonate K, 14 days apart. The strains differed from each other by 24 SNPs indicating potential microevolution following infection.

The outlying single isolate *C. sakazakii* 767 was isolated from neonate H, who died of meningitis (Fig. 3, shaded green). This strain differed from the index strain by 38 SNPs, therefore from the SNP typing and tree topology, it seems likely that this strain differs from the other isolates in cluster 2 due to microevolution from the first group.

The third group within cluster 2 consisted of three strains (705, 706,707; Fig. 3, shaded yellow) was distant to the reference strain as indicated by tree topology and SNP typing. All of the isolates in this group were obtained only from neonate B. Each of these isolates differed from the index strain by more than 300 SNPs. This observation is in agreement with Caubilla-Barron et al. where these strains formed a sub-cluster (differing by one band) within the pulsotype 2 [1]. SNP typing showed that the three isolates differed from each other by a maximum of 16 SNPs; since these three isolates were isolated from neonate B at different time points, the SNP differences between them could be the result of a microevolution. Due to the distance of the group 3 strains from the index strain and other isolates in cluster 2, we believe that these strains indicate that neonate B may have been infected from a different source of *C. sakazakii* CC4 strains than the other neonates.

It is interesting to note that strain 703 from genotype cluster 1, and the third group of cluster 2 isolates (705, 706 and 707) were all isolated from neonate B. However the strains in the two clusters were entirely different (~70,000 SNPs; data not presented) indicating that neonate B was co-infected *C. sakazakii* isolates belonging to different genotype clusters and clonal complexes (CC12 and CC4).

### SNP typing of cluster 3

**Fig. 4:**
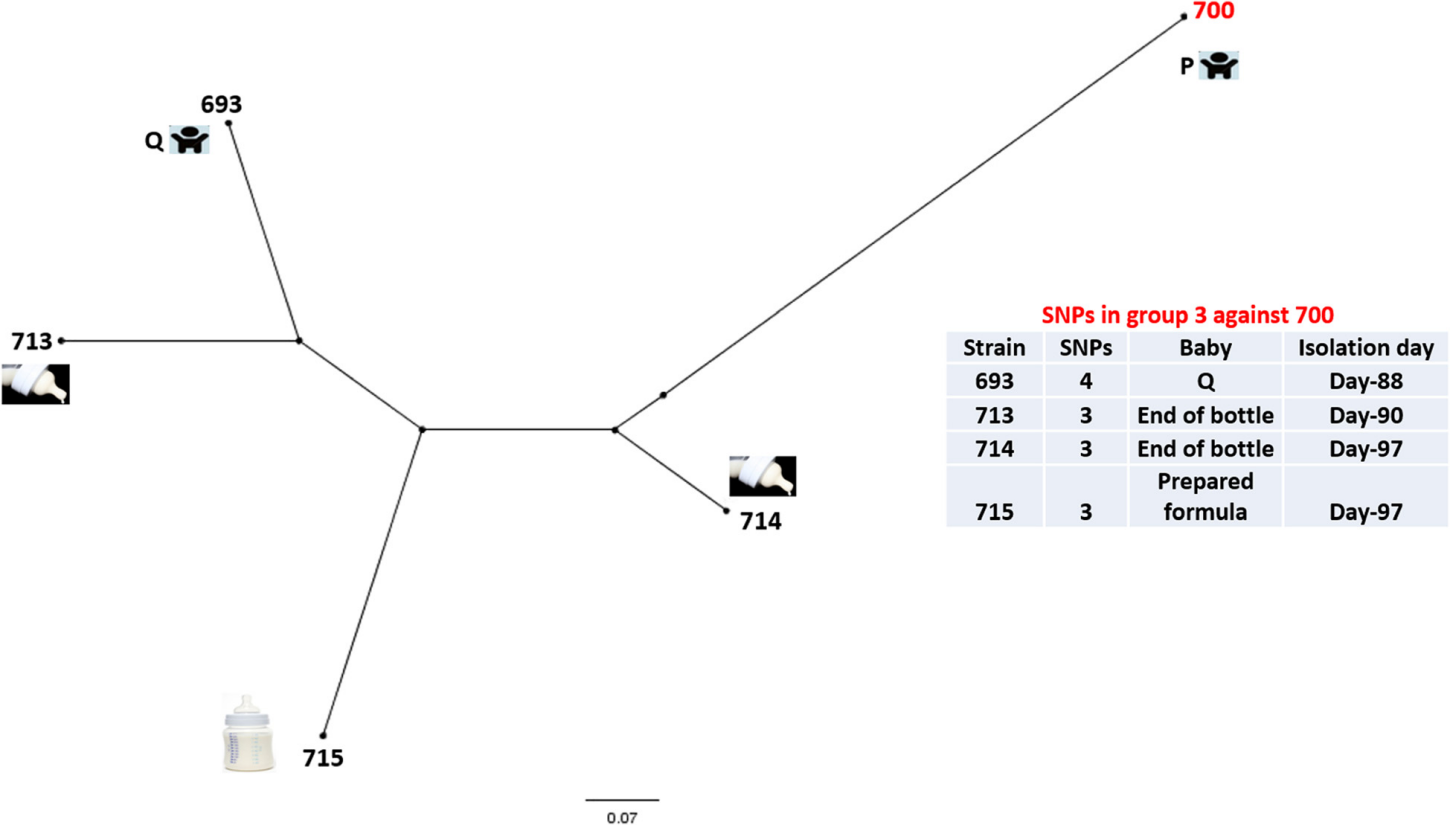
SNP phylogeny of the cluster strains. The SNPS were called using SMALT and SAMtools to generate the VCF files which were filtered using VCFTools to include only SNPs with minimum quality score of 30, minimum depth of 8, and minimum allele frequency of 0.90. The SNPs in the cluster were concatenated and used to create a maximum likelihood phylogeny. The red font colour indicates the reference strain. The tree was rooted to midpoint. The scale bar indicates the rate of nucleotide differences per sequence site

Five of the isolates belonging to cluster 3 were isolated between 15th June and 27th June (Table 1). Two of the isolates (700, 693) were obtained from neonates P and Q, the former had moderate digestive problems while the later was asymptomatic. Of the remaining 3 isolates, 2 isolates (713, 714) were obtained from the end of the bottle while one isolate (715) was obtained from unused prepared formula. The date of their isolation overlapped with the isolates in clusters 1 and 2. All isolates were *C. sakazakii* CC13 and were obtained between days 85 and 97 of the first reported case in the NICU (Table 1). The earliest isolate within this cluster, *C. sakazakii* 700, was used as the index strain to call SNPs in the remaining four isolates. The SNP typing and tree topology of the isolates obtained from neonates P and Q, and isolates obtained from both the end of the bottle and prepared formula indicated that all of these strains were almost identical to the index strain with a maximum of 4 SNPs difference between the isolates (Fig. 4). This observation indicates that the potential source of the neonate infection or colonization was prepared formula. Whether the prepared formula was contaminated because of intrinsic contamination of PIF or was due to contamination from a different source such as water, utensils used to prepare formula feed, or carer’s hands remains unclear. It is known that prepared feeds were temperature abused [1].

### Cluster 4 strain 716

The cluster 4 strain *C. sakazakii* 716 was isolated for unopened tin of PIF. This strain belongs to MLST CC1 which has frequently been associated with infant formula [2]. However in this study no neonatal isolates were recovered which matched strain 716. It is plausible that such further outbreaks were avoided due to improved control measures. At the time of the outbreaks, the NICU feeding practice was to reconstitute formula every 24 h and administer the formula over a 4 to 6 h period by automated infusion using an enteral syringe at room temperature. After the outbreak, internal recommendations were made to chill the enteral feeding syringe during use and to change the syringe and syringe end every 3 h.

## Conclusion

The present study was aimed at the genomic analysis of a *C. sakazakii* outbreak in 1994 that lasted over 3 months in a French NICU. The whole genome phylogeny indicated at least four distinct clusters for the sequenced *C. sakazakii* strains. Each cluster was composed of isolates belonging to different sequence type; cluster 1:ST12, cluster 2:CC4, cluster 3:CC13, and cluster 4:CC1. This is in agreement with Forsythe et al. [2] who showed that 7-loci MLST, rMLST and COG-MLST analysis agreed with whole genome phylogeny. The observation was also in agreement with the previous PFGE profile analysis [1], supporting the continued general usefulness of PFGE to obtain an overall perspective in an outbreak. However, SNP analysis has proven to be more discriminatory as it provides the best resolution at the DNA level [15, 16].

Genomic examination at the SNP level across different *C. sakazakii* clusters confirmed that the clusters were completely unrelated. Moreover, SNP analysis was undertaken to analyse the strain relatedness within each cluster independently of the other clusters. SNP analysis of cluster 1, 2 and 3 revealed the likelihood of a recent a common ancestor resident in the hospital environment. This is evidenced by the small number of SNP differences within each cluster. The values being 64 and 4 nucleotide differences for clusters 1 and 3, and 38 and 10 nucleotides for the two subgroups in cluster 2; Table 1, Figs. 2, 3 and 4.

Another important observation in this study was that different *C. sakazakii* sequence types which were from different genotype cluster groups*,* colonized the same neonates (B, C, D) indicating the ability of *C. sakazakii* strains to co-exist. These isolates from two different clusters were highly divergent as indicated by SNP differences (>70,000 SNPs) suggesting that they were acquired simultaneously from a common source composed of diverse *C. sakazakii* isolates. Whether co-existence of *C. sakazakii* is linked with neonatal infections warrants further investigation and has previously been reported [5]. Although, on this occasion, neonate C, who was colonized by isolates 708 and 709 of cluster 1 and 2 respectively, was asymptomatic.

Improved practices in the manufacturing and preparation in addition to improved personal hygiene are essential to prevent the growth and transmission of this neonatal health associated pathogen.

## Methods

### Bacterial strains

A total of 26 *C. sakazakii* genomes were analysed in this study (Table 1). These represented the strains from the previously assigned four pulsotypes [1]. Since three strains 716, 717 and 718 forming pulsotype 4 in the PFGE profile had been isolated from an un-opened can of the infant formula on the same day, it was assumed that these are multiple isolates of the same strain and hence only one isolate (716) was sequenced from this pulsotype [1].

### Genome sequencing

Bacterial DNA was extracted from 1-day old cultures using GenElute™ bacterial genome kit (Sigma Aldrich®, USA). The genome sequences of *C. sakazakii* strains were generated on an Illumina MiSeq using v3 chemistry and 300 bp paired end reads using dual indexed Nextera XT libraries. The mean insert size was around 250–300 bp. The whole genome shotgun projects for these were deposited at Genbank and their accession numbers are given in Table 1.

### Genome assembly and annotation

*De novo* assembly was performed using Velvet (version 1.2.09) [21] and improved using the Post Assembly Genome Improvement (PAGIT) suite of programmes [22]. The finished genome of *C. sakazakii* SP291 [Genbank accessions CP004091-4] was used as a reference for contig reordering. The genomes were annotated using the prokaryotic genome annotation system (PROKKA) [23].

### Phylogenetic analysis

The genome sequences of 26 *C. sakazakii* genomes (Table 1) were aligned using Mugsy [17] and the core genome extracted as described previously [24–26]. Maximum likelihood phylogeny was then reconstructed using RAxML with the GTR-gamma model [27] and the resulting trees visualised and annotated using Figtree (http://tree.bio.ed.ac.uk/software/figtree/). All genomes were uploaded to the Bacterial Isolate Genome Sequence Database (BIGSdb) supported *Cronobacter* PubMLST database [2, 28]; http://pubmlst.org/cronobacter/.

### Single nucleotide polymorphism analysis

Single Nucleotide Polymorphism (SNP) analysis was performed on sequenced *C. sakazakii* strains belonging to clusters 1, 2 and 3 (Table 1) using SMALT and SAMtools [29, 30]. The SNP calling was done independently for each cluster using the earliest isolate in each cluster as the index strain. The resulting VCF files were filtered using VCFTools to include only SNPs with minimum quality score of 30, minimum depth of 8.0, and minimum allele frequency of 0.90 [24–26]. The SNP calls reflected both synonymous and non-synonymous changes (Additional file 1: Table S1). SNPs in each cluster were concatenated and used to create a maximum likelihood phylogeny. The resulting trees were visualised and annotated using Figtree.

### Ethics statement

Isolates from this study were obtained by culturing archives stock isolates. All clinical data are taken from a previous publication [1].

## Additional file

Additional file 1: Table S1. SNP analysis of strains giving co-ordinate and substitution for each isolate relative to the reference strain. (XLSX 35 kb)

## Abbreviations

CC: Clonal complex
PAGIT: Post assembly genome improvement toolkit
ST: Sequence type
SNP: Single nucleotide polymorphism
VCF: Variant call format
